# Selective Serotonin Reuptake Inhibitors for Children with Autism Spectrum Disorder: A Systematic Review and Meta-Analysis

**DOI:** 10.1177/11795565261442820

**Published:** 2026-06-09

**Authors:** Elisabetta Trinari, Noella Juliana Noronha, Davide Papola, Tahira Devji, Tamara Navarro, Olaf Kraus de Camargo, Alfonso Iorio

**Affiliations:** 1 Department of Pediatrics, McMaster University, Hamilton, ON, Canada; 2 Department of Health Research Methods, Evidence and Impact, McMaster University, Hamilton, ON, Canada; 3 WHO Collaborating Centre for Research and Training in Mental Health and Service Evaluation, Department of Neuroscience, Biomedicine and Movement Sciences Section of Psychiatry, University of Verona, Italy; 4 Department of Global Health and Social Medicine, Harvard Medical School, Boston, MA, USA

**Keywords:** autism, pharmacology, SSRI, GRADE method

## Abstract

**Background:**

Effects of SSRI for symptoms of ASD and comorbid conditions are uncertain. We conducted a systematic review and meta-analyses to determine the efficacy and safety of SSRIs in children with ASD on 8 clinically relevant outcomes.

**Methods:**

We searched 7 databases to retrieve RCT of SSRIs versus placebo in children with ASD. Two authors independently extracted data, assessed risk of bias and rated the certainty of evidence using the GRADE method.

**Results:**

Seven RCTs with a total of 606 participants were included. The evidence is very uncertain on the effect of SSRIs on restricted repetitive behaviors and anxiety symptoms in children with ASD. There is low certainty of the evidence suggesting that SSRIs result in little to no effect on obsessive-compulsive symptoms and disruptive behaviors, and there is moderate certainty of the evidence that SSRIs likely result in little to no difference in global functioning and a slight increase in adverse events. No studies evaluated depressive symptoms.

**Conclusions:**

The number of studies on this population remains limited. The current body of evidence shows no benefit of the use of SSRIs in children with ASD. Risk/benefit assessment should be carefully considered on an individual basis until further evidence is made available.

**Protocol registration number:**

PROSPERO-CRD42020169836

**Lay Abstract:**

**Efficacy of SSRIs in children with ASD**

**Do SSRIs Help Children with Autism?**

The scope of this systematic review of the literature was to identify if medications called SSRIs (Selective Serotonin Reuptake Inhibitors) are helpful and safe for children with Autism Spectrum Disorder (ASD). These medicines are sometimes used to treat anxiety, obsessive-compulsive behaviors, or other symptoms.

**What Did the Study Look At?**

The researchers carefully examined seven studies that included 606 children with ASD. They compared the effects of SSRIs to a placebo (a pill with no active medicine).

**What Did They Find?**

The researchers found that there is not enough data to confidently judge the effect of SSRI on restricted-repetitive behaviors and anxiety symptoms in children with ASD.

Data available shows that SSRIs likely don’t improve obsessive-compulsive symptoms or disruptive behaviors in children with ASD.

SSRIs probably don’t make a noticeable difference in overall daily functioning in children with ASD.

Children taking SSRIs might experience a small increase in side effects like upset stomach or sleep problems.

No studies looked at how SSRIs affect depression in children with ASD.

**What Does This Mean for Families?**

Right now, there isn’t strong evidence to support using SSRIs for children with ASD. Every child is unique, so if you’re considering this medication, it’s essential to weigh the possible risks and benefits with your doctor.

More research is needed to provide better guidance for families and physicians.

## Introduction

### Description of the Condition

Autism Spectrum Disorder (ASD) is an umbrella term defining a developmental disability characterized by the presence of 2 core symptoms: *(1) difficulties in social communication and social interaction*; and *(2) restricted repetitive behaviors, interests or activities*, according to the definition provided by the Diagnostic and Statistical Manual of Mental Disorder – Fifth Edition (DSM-V)^1^ and similarly by the International Classification of Disorders – Eleventh Edition (ICD-11^2^).

Although the etiology of ASD is complex and multifactorial, genetic factors are recognized to play a predominant role in its development. Monozygotic twin studies have demonstrated high heritability estimates ranging from 60% to 90%,^3^ and a large population-based study has confirmed that the majority of ASD liability is attributable to genetic influences.^4^ In addition, various environmental determinants, including prenatal, perinatal, and postnatal factors, have been implicated in ASD risk,^5,6^ although their specific mechanisms and relative contributions to ASD phenotypes remain the focus of ongoing research.^7^

Patients affected by ASD may exhibit different levels of severity and heterogeneous clinical presentations. This definition, introduced in 2013, has encompassed conditions previously identified as Autistic Disorder, Asperger’s Disorder, Pervasive Developmental Disorder Not Otherwise Specified (according to the DSM-IV^8^) and Childhood Autism, Asperger Syndrome, Pervasive Developmental Disorder Unspecified (according to the ICD-10 Revised^9^). In 2014, the prevalence of ASD in the United States was estimated as 1 in every 59 children aged 8 years,^10^ with worldwide estimates around 1% to 2%,^11,12^ and it is 4 times more common in males than females.

The prevalence of co-occurring mental health conditions is substantially higher among individuals with ASD, with up to 70% presenting with at least 1 psychiatric comorbidity.^13^

In this context, supporting families is essential, as parent-focused interventions can improve outcomes and help mitigate caregiver burden.^14^

Educational and behavioral interventions that are well-structured, delivered one-to-one and intensively applied within the first 6 years of age have shown to be beneficial in affecting language and cognition.^15^ Unfortunately, the quality of existing evidence is low, and their implementation is often impeded because these techniques are expensive, time-consuming, and they are not always available or reimbursable.^16^

As a result, pharmacotherapy is widely used^17^ although evidence only supports the treatment of specific co-occurring symptoms or behaviors, such as aggressivity/irritability and hyperactivity. Currently, risperidone and aripiprazole are the only 2 medications that have shown to be effective^18^ and approved to treat severe problem behaviors that occur in about 20% to 30%^19,20^ of children with ASD. Similarly, methylphenidate,^21^ atomoxetine^22^ and guanfacine^23^ can be beneficial for children with an additional diagnosis of Attention Deficit Hyperactivity Disorder (ADHD), occurring in about 28%^24^ of children with ASD.

### Description of the Intervention and How It Might Work

On the other hand, Selective Serotonin Reuptake Inhibitors (SSRIs) are a class of medication frequently used in clinical practice in children with ASD although the evidence base for their efficacy is still inconclusive. Various SSRIs have been approved by the Food and Drug Administration (USA) for the treatment of major depressive disorder and obsessive-compulsive disorder in non-ASD children, with minor variations of indications/dosage among other countries. In children with ASD, SSRIs are often used off-label in an attempt to reduce the frequency and severity of core symptoms, such as repetitive restrictive behaviors (RRBs), or to improve comorbid symptoms, such as anxiety. Two large population-based studies demonstrated the high frequency of polypharmacy in the ASD population, sitting at around 30%, and confirmed that SSRIs account for a relevant percentage of prescriptions (5.6% for fluoxetine and 5.5% for sertraline) with an increasing trend over the years^25,26^

### Why It Is Important To Do This Review

To date, a Cochrane Systematic review published in 2013^27^ has examined the efficacy of 4 SSRIs (fluoxetine, fluvoxamine, fenfluramine and citalopram) in both adult and children populations. The review included 9 randomized controlled trials (RCTs) with a total of 320 adults and children and explored numerous outcomes. Meta-analysis was possible only for 1 single outcome, global functioning, measured with the Clinical Global Impression – Improvement scale (CGI-I). The interest in this topic, however, has remained high: a few additional RCTs in the pediatric populations were published in 2019^28-30^ and new randomized studies are currently ongoing. Two recent systematic reviews and meta-analyses^31,32^ evaluated the efficacy of different treatments on RRBs in pediatric and adult patients with ASD and concluded that SSRIs were not associated with a benefit on RRBs compared to placebo. These reviews, however, did not evaluate other patient-important outcomes, only 1 ^31^ reported a risk of bias assessment of the included studies, and neither of them applied the Grading of Recommendations Assessment, Development and Evaluation (GRADE) method to rate the certainty of the evidence.

An updated and methodologically rigorous systematic review and meta-analysis of RCTs comparing SSRIs versus placebo in pediatric patients with ASD was deemed necessary to gain a better understanding of the risks and benefits of this treatment in this specific population and to inform decision-making with the latest best available evidence, considering the large use of these medications. To address this gap, we focused on important patient outcomes for which there is either a plausible or already demonstrated mechanism of action in other populations. We included outcomes reported in the previous Cochrane review,^27^ related to the pediatric autistic population, as well as well-known outcomes of SSRIs in neurotypical children, like anxiety and depression.^33,34^ We contacted the authors to retrieve additional unpublished data and applied strict methodology to appraise the evidence. We avoided calculating standardized mean differences for various measures assessing similar but not equivalent constructs to warrant transparency in reporting and improve the communication of the results. Finally, we reported Minimal Important Differences for each one of the continuous outcomes to allow a clear interpretation of our findings.

## Objectives

We aim to address 2 key questions in this systematic review: (1) what are the benefits of SSRIs in the pediatric population with ASD? and (2) what are the adverse events (AEs) of SSRIs in children/adolescents with ASD?

## Methods

We followed the Preferred Reporting Items for Systematic Reviews and Meta-Analyses statement^35^ (PRISMA) when conducting and reporting this prospectively registered systematic review.

### Data Sources and Searches

We searched MEDLINE, EMBASE, CINAHL, PsycINFO and The Cochrane Central Register of Controlled Trials (CENTRAL) from inception to the first week of February 2020. The last updated search on MEDLINE, EMBASE, CINAHL and PsycINFO was conducted in January 2024. We did not apply any restrictions based on language, age of participants, publication status or time of publication. Detailed search strategies used for this review are reported in Supplemental Appendix 1. In MEDLINE (Ovid) the subject-specific search strategy was combined with the Cochrane highly sensitive search strategy for identifying randomized trials,^36^ and appropriate adaptations were also applied to the other databases as well.^37,38^

We reviewed the reference lists of the included trials and any relevant review articles retrieved from the electronic searches to identify any other potentially relevant trials. Efforts were also made to identify unpublished studies, such as those presented only in abstracts. Moreover, we identified ongoing trials using the World Health Organization (WHO) International Trials Registry Platform, Current Controlled Trials (ICTRP) and the Clinical.Trials.gov (CT.gov). Authors of trials were contacted by email to request unpublished data when necessary. A final search on CT.gov was repeated on March 4th, 2024, to identify new ongoing relevant studies (Supplemental Appendix 4).

### Study Selection

We included RCTs comparing SSRIs versus placebo in children and adolescents (up to 18 years) with a diagnosis of ASD explicitly made according to DSM-V criteria (or equivalent previous definitions according to DSM-IV or ICD-10). We included trials evaluating an oral SSRI, regardless of the compound, dosage, and frequency of administration. Cross-over studies were also eligible for inclusion when an adequate duration of the washout period was reported (considering half-life of the compound and possible carryover effects). Trials including patients with Rett Syndrome or Childhood Disintegrative Disorder were excluded from this review, considering the prognosis, progressive course of disease and potential differences in response to psychoactive treatments.

Two review authors (ET and NJN) independently screened titles and abstracts of articles retrieved by the searches in duplicate. Studies were initially categorized into “possibly relevant” (when meeting the inclusion criteria or when not possible to determine inclusion from title/abstract) and into “excluded” (when clearly not meeting the inclusion criteria). One review author (ET) retrieved copies of all possibly relevant studies. Two review authors (ET and NJN) then independently and in duplicate reviewed the full text of all retrieved studies against the inclusion and exclusion criteria, with disagreements resolved through discussion.

### Outcomes

The review addressed the following patient/parent important outcomes^39^: restricted repetitive behaviors, obsessive-compulsive symptoms, anxiety symptoms, depressive symptoms, disruptive behaviors, global functioning, quality of life of patient/family, withdrawals due to adverse events (AEs), any AEs.

### Data Extraction and Quality Assessment

Two review authors (ET, NJN) independently extracted key participant and intervention characteristics, and data on efficacy outcomes and adverse events using pre-developed data extraction forms in duplicate. Reviewers abstracted characteristics of eligible studies, including study design, duration of study, number of centers, study settings, number of patients, age, gender, diagnostic criteria, severity of condition, Intellectual Quotient (IQ), inclusion/exclusion criteria, genetic diagnosis, relevant comorbidities, intervention, comparator, concomitant and previous medications, excluded medications, withdrawals and reasons for discontinuation, efficacy and safety outcomes of interest. We extracted outcome data only when trials measured the outcome of interest with a validated instrument previously described in a peer-review journal and not modified by the authors. Disagreements were resolved by discussion, or if required, by a third author (AI). We attempted to locate the protocol of each included study and contacted the authors of included studies to provide additional data when necessary. After completing data extraction, we contacted authors again to confirm the accuracy of extracted data and we received feedback from 3 of them.^28,30,40^

Two review authors (ET, NJN) independently assessed the risk of bias of included RCTs in duplicate. Risk of bias assessments were performed for each specific outcome using the Cochrane Risk of Bias tool – RoB 2.0 tool,^41^ which addresses the following methodological domains: bias arising from randomization process, bias due to deviations from intended interventions, bias due to missing outcome data, bias in measurement of the outcome and bias in selection of the reported result. The risk of bias judgment for each domain is assessed as “low risk,” “high risk” or “some concerns.” Any disagreements in the assessments were resolved by consensus or adjudication by a third review author (AI) when necessary.

### Data Synthesis and Analysis

We performed meta-analysis summarizing dichotomous outcomes using risk ratio (RRs) and Risk Difference, with corresponding 95% confidence intervals (CIs). We calculated the absolute estimates of effect by applying relative risk estimates to a baseline risk, for which we pooled control group event rates from the included RCTs. Continuous outcomes were summarized using mean differences (MD) of post scores with corresponding 95% CIs.

Minimal important differences (MIDs) were used to interpret the mean differences between groups for continuous outcomes. We searched the PROMID (Patient Reported Outcomes Minimal Important Difference) database,^42^ a comprehensive catalog of MIDs for patient-reported outcome measures to retrieve empirically derived MIDs from published anchor-based studies.^43^ We could only identify a single MID estimate for the Children’s Yale-Brown Obsessive Compulsive Scales modified for pervasive developmental disorders (CYBOCS-PDD^44^ rating from 0 to 20, lower score better outcomes; MID: 2 points).^28^ In the absence of other MID estimates for the remaining outcome measures of interest, to enhance interpretability of the results, we assumed a 10% reduction in symptoms could represent an acceptable cut-off to appreciate a small but important change (Supplemental Appendix 6). However, this discretionary threshold is not meant to replace a more appropriate anchor-based estimate of the MID for these outcomes^45^ nor to exclude that an accurate MID may be lower or higher than 10%.

We used Review Manager 5 software (RevMan Web, The Cochrane Collaboration, 2019, available at revman.cochrane.org) for the meta-analyses, with the generic inverse-variance random-effects model for continuous outcomes, and Mantel-Haenszel method and random effects for dichotomous outcomes. Forest plots were used for visualization and a two-sided *P*-value < .05 was considered statistically significant. We assessed statistical heterogeneity with the χ^2^ and *I*^2^ statistics.

We planned to perform 3 subgroup analyses based on age (<12 vs 12-18 years), IQ level (⩽70 vs >70) and diagnosis of genetic syndrome. Sensitivity analysis: We planned to run sensitivity analysis based on high quality RCTs.

### Certainty of the Evidence Assessments

Two review authors (ET and DP) assessed the certainty of the estimates of effect (quality of evidence) for each outcome using the Grading of Recommendations Assessment, Development and Evaluation (GRADE) method,^46^ with 1 author (AI) adjudicating the decision in instances where there were discrepancies. We considered limitations in risk of bias, inconsistency, indirectness, imprecision and publication bias. Certainty in the body of evidence was rated as high (we are very confident that the true effect lies close to that of the estimate of the effect); moderate (the true effect is likely to be close to the estimate of the effect, but there is a possibility that it is substantially different); low (the true effect may be substantially different from the estimate of the effect); or very low (the true effect is likely to be substantially different from the estimate of effect).^47^ GRADEpro software was used to prepare the Summary of Findings tables, which summarizes the certainty of evidence and the magnitude of effect of the intervention examined for all outcomes.^48,49^

## Results

**Figure 1.**
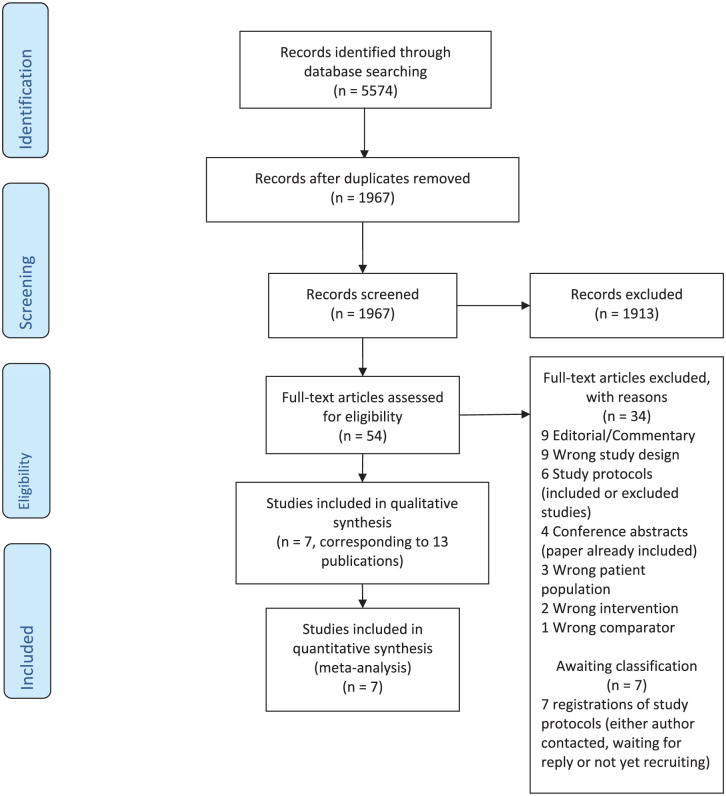
PRISMA flow-chart.

We screened a total of 1893 unique records (after 661 duplicates were removed) identified from the 5 electronic databases. No additional records were identified through other sources. Details of the process of screening and selecting studies for inclusion in the review are presented in Figure 1. Overall, there were a total of 7 included trials (corresponding to 13 publications), 34 excluded articles, and 5 registered protocols awaiting classification. Details on trials excluded at full-text and on studies awaiting classification are reported respectively in Supplemental Appendices 2 and 4.

Of the 7 included studies, 3 studies tested fluoxetine^28,29,50^, 1 citalopram,^51^ 2 sertraline^30,40^ and 1 fluvoxamine,^52^ and the comparator was placebo in each case. Five studies used a parallel design,^28-30,40,51^ and 2 a crossover design.^50,52^ Both studies with crossover designs observed a washout period compatible with the recommended length for the specific SSRI. However, because of a possible period effect in the Hollander et al^50^ study, we pulled data in the meta-analysis only from the first period of treatment. No data were extracted from the Sugie et al^52^ study because results were only presented for genotypes rather than as treatment-placebo comparison. Authors from 3^28,30,40^ studies provided us with additional unpublished data. The Greiss Hess et al^40^ study included participants with Fragile X, however, we considered only the subgroup of patients with ASD. Unpublished data were collected on global functioning (dichotomous^28,30,40^ and continuous results^40^), disruptive behaviours^30^ and adverse events.^40^

Across the included trials, the primary outcome was identified as obsessive-compulsive symptoms in 3 studies,^28,29,50^ global functioning in 2 studies,^40,51^ and expressive language in 2 studies.^30,40^ One study did not specify a primary outcome.^52^

We did not perform the planned subgroup and sensitivity analyses because of the limited data available for meta-analysis. To assess publication bias we searched databases for protocols of unpublished studies^53^ but did not construct a funnel plot due to the small number of studies included in the analyses.

Characteristics of included studies are presented in Supplemental Appendix 3. A comparison with the previous systematic review published in 2013^27^ is presented in Supplemental Appendix 7.

### Risk of Bias of Included Studies

**Figure 2.**
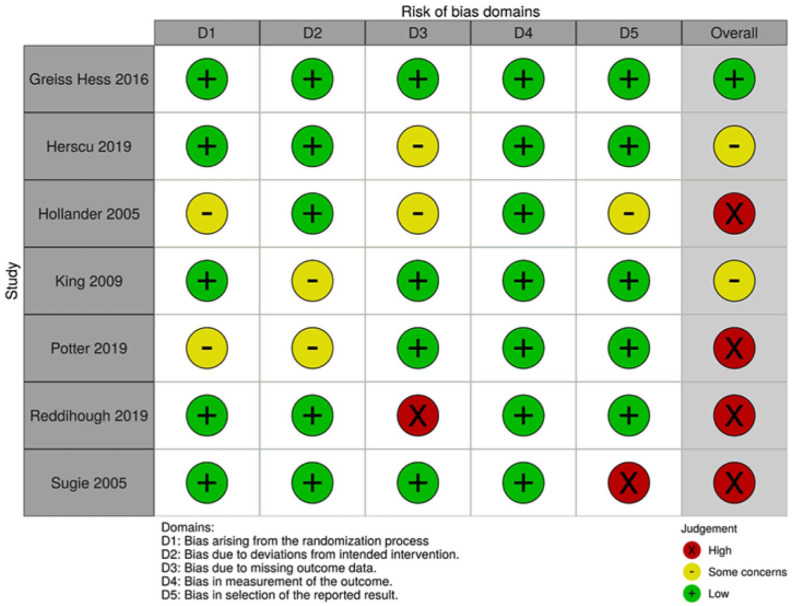
Risk of bias in the included studies for the efficacy outcomes.

**Figure 3.**
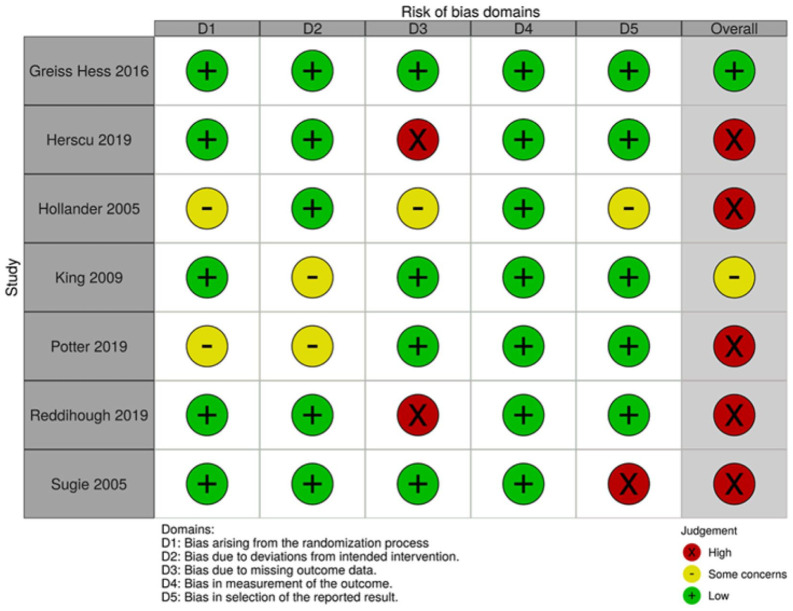
Risk of bias in the included studies for the safety outcomes.

The allocation sequence was randomly generated, and it was concealed in 4 studies with no apparent baseline imbalances.^29,40,51,52^ In one study, there was no information about the randomization process, but no baseline imbalances were reported.^50^ In another study, although random sequence generation was adequate and allocation was concealed, some baseline imbalances were likely due to chance.^28^ Finally, in one study, no details on allocation sequence concealment were reported, and the 2 groups appear unbalanced for various baseline characteristics.^30^ Risk of bias due to deviation from the intended interventions was low in 5 of the 7 included studies,^28,29,40,50,52^ while in one study there were some concerns about the possibility of patients and study personnel being aware of the intervention^51^ and in another study^30^ there were some concerns because Intention-to-Treat (ITT) analysis was conducted for the primary outcomes but not specified for the outcomes of our interest. Risk of bias due to missing outcome data was detected in 3 studies: 2 studies had differential and high dropout rates that led to some concerns^29^ and high risk^28^; for 1 study^50^ there were some concerns because of a 11% of drop-outs that were not included in the ITT analysis and 4 studies were at low risk.^30,40,51,52^ Risk of bias in the measurement of the outcome was judged low in all included studies. Finally, risk of bias for selective reporting was low in 5 studies^28-30,40,51^ for which trial registrations were available as well as details on prespecified outcomes. Whereas, for 1 study^50^ the protocol didn’t provide any detail on outcomes, and for another study the protocol was not available and the analysis reported effectiveness only for genetic subgroups.^52^ Details on risk of bias assessment of the included studies are reported in Supplemental Appendix 3 and associated plots in Figures 2 and 3. When risk of bias has been found to be different for different outcomes in the same study, the judgment has been reported in the specific domain along with the outcome (underscored in Supplemental Appendix 3).

#### Restricted Repetitive Behaviors

Two studies^28,51^ reported restricted repetitive behaviors measured with the Repetitive Behavior Scale-Revised (RBS-R). However, while King et al^51^ reported the mean differences for the 5 subscales, Reddihough et al^28^ reported only the total score. Pooling the total scores was possible by imputing the SD from Reddihough et al.^28^

Pooling data from 2 RCTs^28,51^ including 230 participants, treated for 12 to 16 weeks, showed a MD −3.24 in restricted repetitive behaviors on the RBS-R score with the use of SSRIs (95% CI −14.91, 8.43; *I*^2^ = 74%; MID −12.9 points; Analysis 1). Owing to concerns of serious risk of bias, inconsistency and imprecision, the evidence is very uncertain about the effect SSRIs in children with ASD on Restricted Repetitive Behaviors (Summary of Findings, Table 1).

**Analysis 1.**
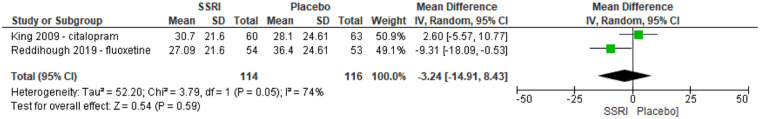
Restricted repetitive behaviors (mean difference for the RBS-R).

**Table 1.** Summary of Findings.

SSRIs compared to placebo in children with ASD						
**Patient or population:** Children with ASD						
**Setting:** Outpatients						
**Intervention:** SSRI						
**Comparison:** Placebo						
Outcomes	Anticipated absolute effects* (95% CI)		Relative effect (95% CI)	No. of participants (studies)	Certainty of the evidence (GRADE)	Comments
	Risk without SSRI	Risk with SSRI				
**Restricted repetitive behaviors**	The mean restricted repetitive behaviors ranged from 28 to 36 points	MD 3.24 points lower (14.91 lower to 8.43 higher)	-	230 (2 RCTs)	⨁◯◯◯	The evidence is very uncertain about the effect SSRIs in children with ASD on Restricted Repetitive Behaviors
Assessed with: RBS-R					Very low^a,b,c^	
Scale from: 0 to 129 (worse)						
Follow up: range 12 wk to 16 wk						
**Obsessive-Compulsive symptoms**	The mean obsessive-Compulsive symptoms ranged from 10 to 13 points	MD 0.1 points lower (1.57 lower to 1.37 higher)	-	413 (3 RCTs)	⨁⨁◯◯	SSRIs may result in little to no difference on Obsessions-Compulsions in children with ASD
Assessed with: CYBOCS-PDD					Low^d,e^	
Scale from: 0 to 20 (worse)						
Follow up: range 12 wk to 16 wk						
**Anxiety symptoms**	Studies showed inconsistent effects–an increase and also a reduction in anxiety symptoms			153 (2 RCTs)	⨁◯◯◯	The evidence is very uncertain about the effect of SSRIs in children with ASD on anxiety symptoms
Assessed with: different scales (PAS-R, SCAS)					Very low^c,f,g^	
Follow up: range 16 wk to 26 wk						
**Depressive symptoms** - not reported	-	-	-	-	-	None of the included studies reported any measure of depressive symptoms in children with ASD
**Disruptive behaviors**	The mean disruptive behaviors ranged from 9 to 13 points	MD 0.2 points higher (2 lower to 2.4 higher)	-	276 (3 RCTs)	⨁⨁◯◯	SSRIs may result in little to no difference in disruptive behaviors
Assessed with: ABC-CV irritability subscale					Low^h,i^	
Scale from: 0 to 45						
Follow up: range 12 wk to 26 wk						
**Global functioning**	32 per 100	32 per 100 (23-44)	RR 0.99	491 (5 RCTs)	⨁⨁⨁◯	SSRIs probably result in little to no difference in the number of patients showing an improvement in global functioning
Assessed with: CGI-I (much or very much improved)			(0.73 to 1.36)		Moderate^h^	
Follow up: range 12 wk to 26 wk						
**Quality of life** (parents)	The mean quality of life (parents) was 2.7 points	MD 0 points (0.24 lower to 0.24 higher)	-	155 (1 RCT)	⨁⨁◯◯	SSRIs (fluoxetine) may not increase nor reduce parent’s quality of life
Assessed with: CSQ					Low^i,j^	
Scale from: 0 to 5						
Follow up: 14 wk						
**Discontinuation due to adverse events**	8 per 100	9 per 100 (5-16)	RR 1.14 (0.64 to 2.04)	511 (4 RCTs)	⨁⨁⨁◯	SSRIs probably result in a slight increase of discontinuation due to adverse events
Follow up: range 12 wk to 26 wk					Moderate^d^	
**Any adverse events**	74 per 100	77 per 100 (70-85)	RR 1.05 (0.95 to 1.15)	543 (5 RCTs)	⨁⨁⨁◯	SSRIs probably result in a slight increase in adverse events
Follow up: range 12 wk to 26 wk					Moderate^k^	

Abbreviations: CI, confidence interval; MD, mean difference; RR, risk ratio.

GRADE Working Group grades of evidence.

**High certainty:** We are very confident that the true effect lies close to that of the estimate of the effect.

**Moderate certainty:** We are moderately confident in the effect estimate: The true effect is likely to be close to the estimate of the effect, but there is a possibility that it is substantially different.

**Low certainty:** Our confidence in the effect estimate is limited: The true effect may be substantially different from the estimate of the effect.

**Very low certainty:** We have very little confidence in the effect estimate: The true effect is likely to be substantially different from the estimate of effect.

Explanations.

a Risk of bias across studies mainly due to missing data.

b statistical inconsistency (*I*^2^ = 74%, chi-squared *P* = .05), partially related to different compounds and doses.

c Substantial imprecision due to a small number of participants and wide CIs including appreciable benefit/harm and no effect.

d Risk of bias due to missing data (high drop-out rate in 2 studies accounting for more than 50% of the weight).

e Substantial heterogeneity not due to chance, both qualitative (likely because of different subpopulation and dose) and quantitative (*I*^2^ of 74%, (*P* = .02) and visual inspection).

f Serious concern of risk of bias mainly due to missing data.

g Serious concern of clinical inconsistency related to different ages, different compounds and doses, and different duration of the treatment.

h Risk of bias due to problem with deviation from intended intervention and missing data.

i Serious concerns of imprecision due to the small sample size.

j Serious concerns of indirectness due to selection of a specific subpopulation (only AD with mild severity).

k Serious concern of risk of bias mainly due to missing data.

* **The risk in the intervention group** (and its 95% confidence interval) is based on the assumed risk in the comparison group and the **relative effect** of the intervention (and its 95% CI).

#### Obsessive-Compulsive Symptoms

Four studies^28,29,50,51^ reported measures of obsessive-compulsive behaviors. However, Hollander et al^50^ used a version of the CYBOCS modified by the investigators recording only compulsive symptoms, and therefore was not included in the meta-analysis.

Pooling data from the 3 RCTs^28,29,51^ including 413 participants, treated for 12 to 16 weeks, showed a MD −0.10 in obsessive-compulsive symptoms on the CYBOCS-PDD score with the use of SSRIs (_95%_CI −1.57, 1.37; *I*^2^ = 74%; MID −2 points; Analysis 2). Due to serious concerns of risk of bias and inconsistency, there is low certainty evidence that suggests that use of SSRIs in children with ASD results in little to no difference in obsessive-compulsive symptoms (Summary of Findings, Table 1).

**Analysis 2.**
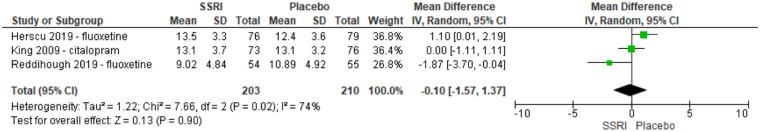
Obsessive-compulsive symptoms (mean difference for the CYBOCS-PDD).

#### Anxiety Symptoms

Two studies^28,30^ reported anxiety symptoms using 2 different scales. Potter et al^30^ reported results from the Preschool Anxiety Scale-Revised (PAS-R) in 47 young children (2-6 years) treated for 26 weeks with a low dose of sertraline (MD 3.34; 95% CI −3.78, 10.46; MID −13.6 point). Reddihough et al^28^ instead used the Spence Children Anxiety Scale (SCAS) and reported the results on 106 older children (7.5-18 years) treated for 16 weeks with a moderate dose of fluoxetine (MD −4.12; 95% CI −10.84, 2.60; MID −11.4 point). Considering the serious concern of risk of bias, inconsistency and imprecision, the evidence is very uncertain about the effect of SSRIs in children with ASD on anxiety symptoms (Summary of Findings, Table 1).

#### Depressive Symptoms

None of the included studies reported any measure of depressive symptoms in children and adolescents with ASD.

#### Disruptive Behaviors

Three studies^28,30,51^ reported maladaptive behaviors using the Aberrant Behavior Checklist–Community Version (ABC-CV) checklist and we reported the irritability subscale that mainly measures disruptive behaviors.

Pooling data from 3 RCTs^28,30,51^ (published and unpublished) including 276 participants treated for 12 to 26 weeks, showed a MD 0.20 in disruptive behaviors measured with the Irritability Subscale of ABC-CV with the use of SSRIs (95% CI −2.00, 2.40; *I*^2^ = 0%; MID −4.5 points; Analysis 3). Because of serious concerns of risk of bias and imprecision, there is low certainty evidence that suggests that use of SSRIs in children with ASD results in little to no difference in disruptive behaviors (Summary of Findings, Table 1).

**Analysis 3.**
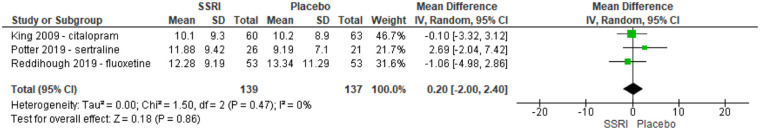
Disruptive behaviors (mean difference for the ABC-Irritability subscale).

#### Global Functioning

All of the included studies used some version of the CGI-I to measure change in global functioning. However, Sugie et al,^52^ in a cross-over trial including 18 patients, presented the CGI scale results for different genotypes only, therefore it was not possible to determine the post-treatment score for the 2 groups.

Collecting additional unpublished data allowed us to pool both dichotomous results (responders defined as “much improved” or “very much improved”), as well as continuous results.

Pooling data from 5 RCTs^28-30,40,51^ including 491 participants, treated for 12 to 26 weeks, showed a RR of 0.99 (95% CI 0.73, 1.36; *I*^2^ = 32%) in the number of responders at the CGI-I score with the use of SSRIs, corresponding to an absolute effect of 0 more participants improving per 100 using SSRIs (from 9 less to 12 more, Analysis 4). Because of the serious concern of risk of bias, there is moderate certainty evidence that suggests that SSRIs in children with ASD probably result in little to no difference in the number of patients showing an improvement in global functioning (Summary of Findings, Table 1).

**Analysis 4.**
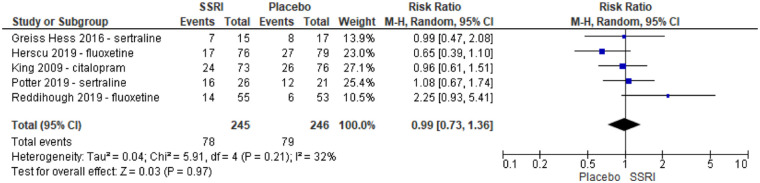
Global functioning (number of responders at the CGI-I).

An additional analysis pooling continuous data on the CGI-I scale from 4 RCTs^28,30,40,50^ including 226 participants showed comparable results (Analysis 5).

**Analysis 5.**
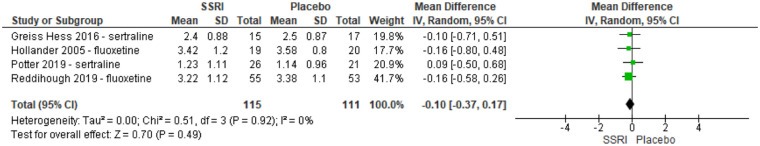
Global functioning (mean difference for the CGI-I).

#### Quality of Life

Only 1^29^ RCT reported quality of life measured with the Caregiver Strain Questionnaire (CSQ) for a group of 121 participants treated for 14 weeks (MD 0.00; 95% CI −0.27, 0.27; MID −0.5 point). Because of serious concerns of indirectness and imprecision, there is low certainty evidence that suggests that SSRIs may not increase nor reduce parent’s quality of life (Summary of Findings, Table 1).

#### Adverse Events

##### Withdrawal Due to Adverse Events

Three studies^28,29,51^ reported the number of withdrawals due to adverse events and 1 study^29^ provided us with unpublished data (Analysis 6a). One additional study reported no withdrawal due to adverse events in both arms (Greiss Hess et al^40^ unpublished data for the subgroup of 32 participants with ASD). Similarly, there were no discontinuation due to adverse events reported in the publication of the 2 cross-over studies: Hollander et al^50^ (44 participants) and Sugie et al^52^ (19 participants). Those results, excluded from the meta-analysis of RRs, were included in an additional analysis of Risk Difference (Analysis 6b) obtaining comparable results.

**Analysis 6a.**
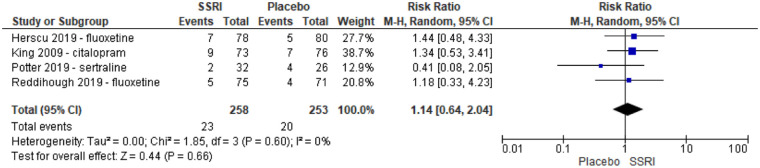
Withdrawal due to adverse events (RR).

**Analysis 6b.**
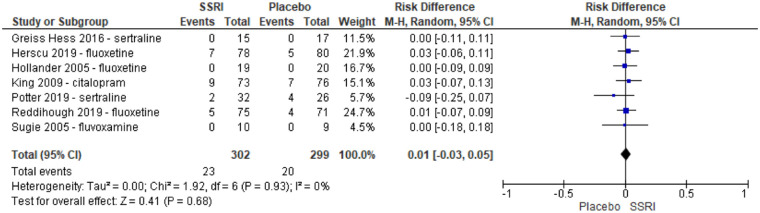
Withdrawal due to adverse events (RD).

Pooling data from 4 RCTs^28-30,51^ including 511 participants, treated for 12 to 26 weeks, showed a RR of 1.14 (95% CI 0.64 to 2.04, *I*^2^ = 0%) in the number of withdrawals due to AEs with the use of SSRIs, corresponding to an absolute effect of 1 more event per 100 people using SSRIs (from 3 less to 8 more). Because of serious concern of risk of bias, there is moderate certainty evidence that suggests that SSRIs probably result in a slight increase of discontinuation due to adverse events (Summary of Findings, Table 1).

##### Any Adverse Events

Adverse events were inconsistently described among studies. The most commonly reported adverse events were gastrointestinal disorders, activation/hyperactivity, sleep disorders, and agitation/irritability, mainly of mild or moderate level of severity. Among the few serious adverse events reported there were 4 cases of suicidal ideation, 3 with placebo^28,50^ and 1 with SSRI,^29^ and 1 case of seizure with SSRI^51^ requiring emergency hospitalization.

Pooling data from 5 RCTs^28-30,40,51^ (both published and unpublished data) including 543 participants, treated for 12 to 26 weeks, showed a RR of 1.05 (95% CI 0.95, 1.15; *I*^2^ = 0%; Analysis 7) in the number of participants experiencing at least 1 adverse event with the use of SSRIs, corresponding to an absolute effect of 3 more participants experiencing adverse events per 100 people using SSRIs (from 4 less to 11 more). Because of serious concern of risk of bias, there is moderate certainty evidence that suggests that SSRIs in children with ASD probably result in a slight increase of adverse events (Summary of Findings, Table 1).

**Analysis 7.**
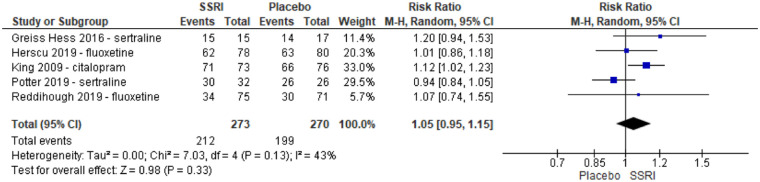
Participants experiencing at least 1 adverse event (RR).

## Discussion

### Main Findings

This systematic review and meta-analysis shows that the number of studies conducted on the use of SSRIs in ASD remains limited, however among the few rigorous pediatric trials completed to date, findings are consistent. Compared to the previous review,^27^ 4 additional studies with rigorous methodology were conducted in the pediatric population.

Overall, the current evidence does not suggest a benefit of SSRIs for patient- and parent-important outcomes in children and adolescents with ASD. The certainty of the evidence is not homogeneous across outcomes. There is very low certainty of the evidence for restricted repetitive behaviors and anxiety symptoms, and low certainty of evidence for obsessive-compulsive symptoms, disruptive behaviors and quality of life, suggesting little to no difference with SSRIs use. In contrast, there is moderate certainty evidence that SSRIs probably result in no difference in global functioning and in a slight increase in adverse events. Notably, no RCTs are testing the efficacy of SSRIs for depressive symptoms in children with ASD.

### Interpretation and Applicability

Despite the lack of conclusive evidence from randomized clinical trials supporting the use of SSRIs in the pediatric ASD population, SSRIs continue to be widely prescribed in clinical practice. Several factors may contribute to this ongoing use and persistent evidence-practice gap.

The scarcity of pharmacological interventions with established efficacy for ASD-associated symptoms often leaves clinicians relying on off-label treatments. SSRIs, in particular, may be chosen because of clinicians’ familiarity, their known effect on anxiety and OCD symptoms in non-autistic children, and their generally acceptable tolerability profile. In addition, when symptoms are persistent and impairing, parental expectations and the hope of alleviating difficulties may play a role in prescribing decisions.

Finally, clinicians’ reports of anecdotal or individual patient benefits, although not consistently demonstrated in trials, may further reinforce continued use. These anecdotal findings raise the possibility that SSRIs could be beneficial in a specific subgroup of children and adolescents with ASD characterized by a particular phenotypic or genotypic profile. This hypothesis, however, has yet to be proven, emphasizing the need for rigorous and adequately powered trials.

Our review focused on children and adolescents with ASD, considering age and neurodevelopmental level as potential modifiers of treatment response. Therefore, these findings might not directly extend to adults with ASD, for whom a separate evidence synthesis would be required.

### Methodological Considerations and Limitations

The marked phenotypic and genotypic heterogeneity within Autism Spectrum Disorder creates substantial methodological challenges when designing rigorous intervention trials. Although secondary subgroup analyses are often conducted to identify differential responses, they are typically exploratory and underpowered. Larger RCTs with well-characterized participants would allow more robust effect estimates. Relevant variables such as IQ, language, global functioning, comorbid psychiatric conditions, and associated genetic variances might influence the effect of SSRIs, acting as moderators or possibly as independent prognostic factors, and should be systematically explored.

Careful selection of outcome measures is also critical. Establishing a minimum baseline severity level for target symptoms and using reliable and sensitive measures of change are crucial for evaluating treatment effects. Lastly, differences in dosage and treatment duration may introduce additional heterogeneity.

Although subgroup analyses exploring the impact of some of these factors were planned in this review, they could not be completed due to the paucity and heterogeneity of available evidence.

### Implications for Future Research

Future high-quality trials addressing these additional considerations may improve understanding of whether there is a role for the use of SSRIs in well-defined subgroups of children with ASD. Shared practical guidelines for conducting RCTs in autism may significantly improve the comparability of results and strengthen the resulting conclusions.

## Conclusions

Current randomized evidence does not support a meaningful benefit of SSRIs for key patient- and parent-important outcomes in children and adolescents with ASD and suggests a slight increase in adverse events, with certainty ranging from very low to moderate depending on the outcome. No randomized trials currently address depressive symptoms in this population. Clinical decisions regarding SSRIs use for co-occurring conditions should therefore rely on individualized risk–benefit assessment, indirect evidence from other pediatric populations, and shared decision-making with patients and caregivers, with transparent discussion of the limitations of the current evidence base.

## Supplemental Material

sj-docx-1-pdi-10.1177_11795565261442820 – Supplemental material for Selective Serotonin Reuptake Inhibitors for Children with Autism Spectrum Disorder: A Systematic Review and Meta-Analysis Supplemental material, sj-docx-1-pdi-10.1177_11795565261442820 for Selective Serotonin Reuptake Inhibitors for Children with Autism Spectrum Disorder: A Systematic Review and Meta-Analysis by Elisabetta Trinari, Noella Juliana Noronha, Davide Papola, Tahira Devji, Tamara Navarro, Olaf Kraus de Camargo and Alfonso Iorio in Clinical Medicine Insights: Pediatrics

sj-docx-2-pdi-10.1177_11795565261442820 – Supplemental material for Selective Serotonin Reuptake Inhibitors for Children with Autism Spectrum Disorder: A Systematic Review and Meta-Analysis Supplemental material, sj-docx-2-pdi-10.1177_11795565261442820 for Selective Serotonin Reuptake Inhibitors for Children with Autism Spectrum Disorder: A Systematic Review and Meta-Analysis by Elisabetta Trinari, Noella Juliana Noronha, Davide Papola, Tahira Devji, Tamara Navarro, Olaf Kraus de Camargo and Alfonso Iorio in Clinical Medicine Insights: Pediatrics
