# Supplementary material for: Selective Serotonin Reuptake Inhibitors for Children with Autism Spectrum Disorder: A Systematic Review and Meta-Analysis
Source: Clin Med Insights Pediatr. 2026 Jun 9;20:11795565261442820. doi: 10.1177/11795565261442820 (PMC13254149; doi:10.1177/11795565261442820)
Supplement: sj-docx-1-pdi-10.1177_11795565261442820 – Supplemental material for Selective Serotonin Reuptake Inhibitors for Children with Autism Spectrum Disorder: A Systematic Review and Meta-Analysis [file sj-docx-1-pdi-10.1177_11795565261442820.docx]

# APPENDICES

**APPENDIX 1 – SEARCH STRATEGIES**

**OVID Medline** Epub Ahead of Print, In-Process & Other Non-Indexed Citations, Ovid MEDLINE(R) Daily and Ovid - MEDLINE(R) from 1946

1     exp Child Development Disorders, Pervasive/
2     pervasive development$ disorder$.tw.
3     autis$.tw.
4     asperg$.tw.
5     PDD$.tw.
6     ASD$.tw.
7    (language adj3 delay$).tw.
8    ((communicat$ or speech) adj3 disorder$).tw.
9     childhood [schizophrenia.tw](http://schizophrenia.tw/).
10     kanner$.tw.
11     [autism.mp](http://autism.mp/). or exp Autistic Disorder/
12     1 or 2 or 3 or 4 or 5 or 7 or 8 or 9 or 10 or 11
13     exp Serotonin Uptake Inhibitors/
14     selective serotonin reuptake inhibitor$.tw.
15    (serotonin* and (reuptake or re-uptake) and inhibitor*).mp.
16     SSRI.tw.
17     [5-hydroxytryptamine.tw](http://5-hydroxytryptamine.tw/).
18     5HT.tw.
19     Fluvoxamine/
20     [fluvoxamine.tw](http://fluvoxamine.tw/).
21     Fluoxetine/
22     [fluoxetine.tw](http://fluoxetine.tw/).
23     Paroxetine/
24     [paroxetine.tw](http://paroxetine.tw/).
25     Sertraline/
26     [sertraline.tw](http://sertraline.tw/).
27     Citalopram/
28     [citalopram.tw](http://citalopram.tw/).
29     escitalopram/
30     [escitalopram.tw](http://escitalopram.tw/).
31     13 or 14 or 15 or 16 or 17 or 18 or 19 or 20 or 21 or 22 or 23 or 24 or 25 or 26 or 27 or 28 or 29 or 30 (76399)
32     12 and 31
33     randomized controlled [trial.pt](http://trial.pt/).
34     controlled clinical [trial.pt](http://trial.pt/).
35     randomized.ab.
36     placebo.ab.
37     drug therapy.fs.
38     randomly.ab.
39     trial.ab.
40     groups.ab.
41     33 or 34 or 35 or 36 or 37 or 38 or 39 or 40
42     exp animals/ not humans.sh.
43     41 not 42
44     32 and 43

**Embase** from 1974

1     exp Child Development Disorders, Pervasive/
2     pervasive development$ disorder$.tw.
3     autis$.tw.
4     asperg$.tw.
5     PDD$.tw.
6     ASD$.tw.
7     (language adj3 delay$).tw.
8     ((communicat$ or speech) adj3 disorder$).tw.
9     childhood [schizophrenia.tw](http://schizophrenia.tw/).
10    kanner$.tw.
11    [autism.mp](http://autism.mp/). or exp Autistic Disorder/
12     1 or 2 or 3 or 4 or 5 or 7 or 8 or 9 or 10 or 11
13     exp Serotonin Uptake Inhibitors/
14     selective serotonin reuptake inhibitor$.tw.
15    (serotonin* and (reuptake or re-uptake) and inhibitor*).mp.
16     SSRI.tw.
17     [5-hydroxytryptamine.tw](http://5-hydroxytryptamine.tw/).
18     5HT.tw.
19     Fluvoxamine/
20     [fluvoxamine.tw](http://fluvoxamine.tw/).
21     Fluoxetine/
22     [fluoxetine.tw](http://fluoxetine.tw/).
23     Paroxetine/
24     [paroxetine.tw](http://paroxetine.tw/).
25     Sertraline/
26     [sertraline.tw](http://sertraline.tw/).
27     Citalopram/
28     [citalopram.tw](http://citalopram.tw/).
29     escitalopram/
30     [escitalopram.tw](http://escitalopram.tw/).
31     13 or 14 or 15 or 16 or 17 or 18 or 19 or 20 or 21 or 22 or 23 or 24 or 25 or 26 or 27 or 28 or 29 or 30
32     12 and 31
33     [random.tw](http://random.tw/).
34     clinical [trial.mp](http://trial.mp/).
35     exp health care quality/
36     33 or 34 or 35
37     32 and 36

**Cochrane Central Register of Controlled Trials (CENTRAL), part of the Cochrane Library**

#1 MeSH descriptor: [Developmental Disabilities] explode all trees

#2 (communicat*)

#3 (autis*)

#4 PDD*

#5 ASD*

#6 ("pervasive developmental disorder"):ti,ab,kw

#7 MeSH descriptor: [Asperger Syndrome] explode all trees

#8 MeSH descriptor: [Autism Spectrum Disorder] explode all trees

#9 (kanner):ti,ab,kw

#10 (pervasive next developmental disorder*)

#11 (language near/3 delay*)

#12 speech near/3 disorder*

#13 childhood next schizophrenia

#14 MeSH descriptor: [Child Development Disorders, Pervasive] explode all trees

#15 MeSH descriptor: [Social Behavior Disorders] this term only

#16 MeSH descriptor: [Child Behavior Disorders] this term only

#17 MeSH descriptor: [Speech Disorders] this term only

#18 MeSH descriptor: [Language Development Disorders] this term only

#19 MeSH descriptor: [Communication Disorders] this term only

#20 #1 OR #2 OR #3 OR #4 OR #5 OR #6 OR #7 OR #8 OR #9 OR #10 OR #11 OR #12 OR #13 OR #14 OR #15 OR #16 OR #17 OR #18 OR #19

#21 MeSH descriptor: [Serotonin Uptake Inhibitors] explode all trees

#22 selective serotonin reuptake inhibitors

#23 SSRI

#24 5 hydroxytryptamine

#25 5HT

#26 MeSH descriptor: [Fluvoxamine] explode all trees

#27 fluvoxamine

#28 MeSH descriptor: [Fluoxetine] explode all trees

#29 fluoxetine

#30 MeSH descriptor: [Paroxetine] explode all trees

#31 paroxetine

#32 MeSH descriptor: [Sertraline] explode all trees

#33 sertraline

#34 MeSH descriptor: [Citalopram] explode all trees

#35 citalopram

#36 escitalopram

#37 #21 OR #22 OR #23 OR #24 OR #25 OR #26 OR #27 OR #28 OR #29 OR #30 OR #31 OR #32 OR #33 R #34 OR #35 OR #36

#38 #20 AND #37

**CINAHL** (via EBSCOhost)

S37 S24 AND S38

S36 S25 OR S26 OR S27 OR S28 OR S29 OR S30 OR S31 OR S32 OR S33 OR S34 OR S35 OR S36 OR S37

S35 (MH "Crossover Design") OR (MH "Experimental Studies+") OR (MM "Quantitative Studies") OR (MM "Quasi-Experimental Studies")

S34 (trebl* N3 mask*) or (trebl* N3 blind*)

S33 (doubl* N3 mask*) or (doubl* N3 blind*)

S32 (singl* N3 mask*) or (singl* N3 blind*)

S31 (clinic* N3 trial*) or (control* N3 trial*)

S30 (random* N3 allocat* ) or (random* N3 assign*)

S29 placebo *

S28 random* allocat*

S27 randomis* OR randomiz*

S26 (MH "Randomized Controlled Trials+") OR (MH "Clinical Trials") OR (MM "Double-Blind Studies") OR (MM "Intervention Trials") OR (MM "Triple-Blind Studies") OR (MM "Therapeutic Trials") OR (MM "Single-Blind Studies")

S25 (MM "Random Assignment")

S24 S12 AND S23

S23 S13 OR S14 OR S15 OR S16 OR S17 OR S18 OR S19 OR S20 OR S21 OR S22

S22 "escitalopram"

S21 (MH "Citalopram") OR "citalopram"

S20 (MH "Sertraline Hydrochloride") OR "sertraline"

S19 (MH "Paroxetine") OR "paroxetine"

S18 (MH "Fluoxetine+") OR "fluoxetina"

S17 (MH "Fluvoxamine Maleate") OR "fluvoxamine"

S16 5HT

S15 SSRI

S14 selective serotonine reuptake inhibitor*

S13 (MH "Serotonin Uptake Inhibitors+") OR (MM "Citalopram") OR (MM "Fluoxetine") OR (MM "Fluvoxamine Maleate") OR (MM "Paroxetine") OR (MM "Sertraline Hydrochloride")

S12 S1 OR S2 OR S3 OR S4 OR S5 OR S6 OR S7 OR S8 OR S9 OR S10 OR S11

S11 asperg*

S10 kanner*

S9 communication N3 disorder*

S8 speech N3 disorder*

S7 language N3 delay*

S6 "ASD"

S5 "PDD"

S4 (MH "Asperger Syndrome") OR (MH "Autistic Disorder") OR (MH "Pervasive Developmental Disorder-Not Otherwise Specified") OR (MH "Schizophrenia, Childhood") OR (MH "Child Development Disorders, Pervasive")

S3 (MH "Communicative Disorders+") OR (MM "Language Disorders") OR (MM "Speech Disorders") OR (MH "Compulsive Behavior") OR (MH "Social Behavior Disorders+") OR (MH "Child Development Disorders, Pervasive+")

S2 (MM "Asperger Syndrome") OR (MM "Pervasive Developmental Disorder-Not Otherwise Specified") OR (MM "Schizophrenia, Childhood") OR (MH "Child Development Disorders, Pervasive+")

S1 (MH "Child Development Disorders, Pervasive+")

**PsycINFO**, from 1806

1     pervasive development$ disorder$.tw.
2     autis$.tw.
3     asperg$.tw.
4     PDD$.tw.
5     ASD$.tw.
6     (language adj3 delay$).tw.
7     ((communicat$ or speech) adj3 disorder$).tw.
8     childhood [schizophrenia.tw](http://schizophrenia.tw/).
9     kanner$.tw.
10     [autism.mp](http://autism.mp/). or exp Autistic Disorder/
11     or/1-10
12     selective serotonin reuptake inhibitor$.tw.
13     (serotonin* and (reuptake or re-uptake) and inhibitor*).mp.
14     SSRI.tw.
15     [5-hydroxytryptamine.tw](http://5-hydroxytryptamine.tw/).
16     5HT.tw.
17     Fluvoxamine/
18     [fluvoxamine.tw](http://fluvoxamine.tw/).
19     Fluoxetine/
20     [fluoxetine.tw](http://fluoxetine.tw/).
21     Paroxetine/
22     [paroxetine.tw](http://paroxetine.tw/).
23     Sertraline/
24     [sertraline.tw](http://sertraline.tw/).
25     Citalopram/
26     [citalopram.tw](http://citalopram.tw/).
27     escitalopram/
28     es[citalopram.tw](http://citalopram.tw/).
29     or/12-28
30     11 and 29
31     Treatment Effectiveness Evaluation/
32     exp Treatment Outcomes/
33     Psychotherapeutic Outcomes/
34     PLACEBO/
35     exp Followup Studies/
36     placebo$.tw.
37     random$.tw.
38     comparative stud$.tw.
39     randomi#ed controlled trial$.tw.
40     (clinical adj3 trial$).tw.
41     (research adj3 design).tw.
42     (evaluat$ adj3 stud$).tw.
43     (prospectiv$ adj3 stud$).tw.
44     ((singl$ or doubl$ or trebl$ or tripl$) adj3 (blind$ or mask$)).tw.
45     control$.tw.
46     or/31-45
47     30 and 46

**APPENDIX 2 - LIST OF EXCLUDED STUDIES AT FULL-TEXT**

| ***Studies*** | ***Reason for exclusion*** |
| --- | --- |
| Reinblatt 2009(54) | Wrong population (anxiety disorder) |
| Buchsbaum 2001(55), Vegso 1995(56) | Wrong population (adult population) |
| Albertini 2004(57), Mehlinger 1990(58), Ozbayrak 1997(59), Posey 1999(60) | Wrong design (case report) |
| Award 1996(61), Bishop 2015(62), Peral 1999(63), Todd 1991(64), Belzeaux 2020(65) | Wrong design (not randomized) |
| Chantiluke 2015(66,67) | Wrong intervention (single dose) |
| Anagnostou 2006(68) | Wrong comparator |

Note: Editorials, commentaries, conference abstracts and protocols of excluded trials are not reported here.

**APPENDIX 3 - DETAILED TABLES OF CHARACTERISTICS OF INCLUDED STUDIES AND RISK OF BIAS ASSESSMENT**

| **Herscu 2020, first published on-line in 2019 (published data only)** | | |
| --- | --- | --- |
| Methods | Parallel trial (US, multi-center) | |
| Participants | 158 children randomised, 80 to placebo and 78 to treatment group  (15 discontinued from placebo and 22 from intervention group)  14.6% F  mean age 9.0 ± 3.3, range 5 to 17  Diagnosis: DSM-IV-TR criteria only for Autistic Disorder (Asperger Disorder or PDD-NOS excluded)  At least CYBOCS-PDD score ≥ 10; very severe patients excluded  % of Intellectual Disability not reported | |
| Intervention | Fluoxetine 14 weeks  Flexible titration up to 18mg/day maximum (mean final dose 11.8 mg/day)  Placebo matching active medication | |
| Outcomes  (**in bold outcomes extracted**) | **CYBOCS-PDD (physician rated)**  **CGI-I-AD (dichotomus, physician rated)**  CGI-S-AD (physician rated)  **Caregiver Strain Questionnaire (CSQ)**  **Adverse events** | |
| Notes |  | |
| ***Risk of bias*** | | |
| **Bias** | **Authors’ judgement** | **Support for judgement** |
| Bias arising from randomization process | LOW | randomization using a block size of 4 - treatment group was assigned centrally through the use of an automated clinical trials database- no relevant baseline imbalances |
| Bias due to deviations from intended interventions | LOW | double blind – placebo matched active medication for taste as far as possible - ITT analysis |
| Bias due to missing outcome data | SOME CONCERNS  **(HIGH risk for the adverse events outcomes)** | High differential dropout rate (18% vs 28%) - higher number of lost to follow up in the intervention arm that may depend on the true value, especially for safety outcomes |
| Bias in measurement of the outcome | LOW | double blinded - scoring physician blinded to intervention and also to adverse events |
| Bias in selection of the reported result | LOW | Outcomes pre-specified and analysed in accordance with a plan - protocol publicly available in the trial registry |

Protocol available: <https://clinicaltrials.gov/ct2/show/NCT00515320>

| **Hollander 2005 (published data only)** | | |
| --- | --- | --- |
| Methods | Cross-over (USA-single center) | |
| Participants | N = 44, 39 completed (20 condition-1 and 19 condition-2, 5 lost to follow-up) Children only  30 boys, 9 girls (23% F)  mean age 8.18 ± 3.0, range 5 to 16  Diagnosis: DSM-IV-TR criteria for Autism, PDD-NOS or Asperger Syndrome (12.8%)  59% Intellectual Disability  No required threshold for obsessive-compulsive behaviours | |
| Intervention | Treatment: fluoxetine 8 weeks treatment, 4 weeks wash-out, 8 weeks cross-over  2.5 mg/day up to 0.8 mg/kg/day maximum (mean final dose 9.9±4.35 mg/day)  Placebo matching the active drug | |
| Outcomes  (**in bold outcomes extracted**) | **CY-BOCS (Children’s Yale-Brown Obsessive-Compulsion Scale, compulsion subscale only)**  **CGI-AD (Clinical Global Improvement Scale Adapted to Global Autism) (continuous)**  Global Autism Composite Improvement Measure (composite measure created by the authors)  **Adverse events** (Fluoxetine side effects checklist and Suicidality Subscale of Overt Aggression Scale) | |
| Notes |  | |
| ***Risk of bias*** | | |
| **Bias** | **Authors’ judgement** | **Support for judgement** |
| Bias arising from randomization process | SOME CONCERNS | Random sequence generation and allocation concealment: not stated  No baseline imbalances, 1:1 ratio |
| Bias due to deviations from intended interventions | LOW | "Double blind" and carryover effect likely disappeared |
| Bias due to missing outcome data | SOME CONCERNS | Of 44 patients randomized, 1 was excluded to lack of efficacy and 3 were excluded due to non-compliance (and an additional one due to lost of records)  ITT analysis not possible |
| Bias in measurement of the outcome | LOW | The outcome assessor was blinded to treatment condition and to adverse events |
| Bias in selection of the reported result | SOME CONCERNS | No information available in the protocol about what was planned |

Protocol available: <https://clinicaltrials.gov/ct2/show/NCT00004486?term=fluoxetine&cond=Autism&cntry=US&age=0&draw=2&rank=2>

| **King 2009 (published data only)**  *(King 2013*(69) *Companion paper – post-hoc analysis: no additional data of interest)*  *(Scahill 2012*(70) *Companion paper – no additional data of interest)* | | |
| --- | --- | --- |
| Methods | Parallel trial (USA, multi-centre) | |
| Participants | 149 children randomised, 76 to placebo and 73 to treatment group  (13 withdrew from each group)  14% F  mean age 9.4 ± 3.1, range 5 to 17  Diagnosis: DSM-IV-TR criteria for Autistic Disorder, Asperger Disorder or PDD-NOS  At least moderate severity on CGI-S and at least moderate compulsive behaviours (CYBOCS-PDD ≥8)  39% Intellectual Disability | |
| Intervention | Liquid citalopram 12 weeks  starting 2.5 mg/day up to a maximum of 20 mg/day (mean final dose 16.5±6.5mg)  Placebo matched for smell, taste and viscosity | |
| Outcomes  (**in bold outcomes extracted**) | **CGI-I (dichotomus, clinician-rated)**  **CYBOCS-PDD (clinician-rated)**  Composite measure of the CGI improvement scale and CYBOCS-PDD  **6 subscales of the Repetitive Behaviour Scale (RBS-R) (parent-rated)**  **5 subscales of the Aberrant Behavior Checklist-Community version (ABC-CV)**  **Adverse events** | |
| Notes |  | |
| ***Risk of bias*** | | |
| **Bias** | **Authors’ judgement** | **Support for judgement** |
| Bias arising from randomization process permuted | LOW | Random sequence generation through permuted blocks with randomly varying block sizes stratified by site and by age, allocation distal to investigator, no baseline imbalances |
| Bias due to deviations from intended interventions | SOME CONCERNS | Blinding of the evaluating clinician but no mention of blinding of participants and personnel |
| Bias due to missing outcome data | LOW | Drop outs are substantially balanced between the two groups (17.1% vs 17.8%)  ITT analyses and last observation was carried forward used for missing data |
| Bias in measurement of the outcome | LOW | “The evaluating clinician monitored efficacy and was blinded to adverse events” – “All raters achieved the criterion of within 2 points of the gold standard established by an expert rater” |
| Bias in selection of the reported result | LOW | Results for all outcomes and scales prespecified in the trial registry were available |

Protocol available: <https://clinicaltrials.gov/ct2/show/NCT00086645>

| **Potter 2019 (both published data and data retrieved for authors)** | | |
| --- | --- | --- |
| Methods | Parallel trial (US, Single center) | |
| Participants | 58 children randomised, 26 to placebo and 32 to treatment group (5 discontinued from placebo and 8 from intervention group)  21% F  mean age 4 ± 1, range 2 to 6  Diagnosis: DSM-V criteria for of Autism Spectrum Disorder  (5.2% ASD, 94.8% Autism)  % Intellectual Disability not reported  No Fragile-X  No required threshold for symptoms | |
| Intervention | Liquid sertraline 26 weeks  2.5mg/day under 4y, 5mg/day 4y and older  Placebo matched for appearance and smell | |
| Outcomes  (**in bold outcomes extracted**)  (*in cursive additional data retrieved from authors*) | Mullen Scales of Early Learning - MSEL  **CGI-I (continuous)**, *CGI-I (dichotomus)*  Preschool Language Scales - PLS-5  Visual Analog Scale - VAS  Vinalend II Adaptive Behaviour Composite - VABS-II  **Aberrant Behavior Checklist-Community Version (total score, parent reported)**  *Aberrant Behavior Checklist-Community Version (Irritability subscale)*  **Preschool Anxiety Scale-Revised – PAS-R (total raw score, parent reported)**  Social Responsiveness Scale -SRS  Sensory Processing Measure – Preschool - SPM-P  **Adverse Events** | |
| Notes |  | |
| ***Risk of bias*** | | |
| **Bias** | **Authors’ judgement** | **Support for judgement** |
| Bias arising from randomization process | SOME CONCERN | “The UC Davis Investigational Drug Services independently carried out randomization to sertraline or placebo” – no additional details. Intervention and placebo group numerically unbalanced (32 vs 26 participants) |
| Bias due to deviations from intended interventions | SOME CONCERNS | "Double blind", placebo matching the active drug, ITT conducted for the primary outcome but not specified for the outcomes of our interest |
| Bias due to missing outcome data | LOW | missing outcome data for 6 patients in the intervention arm and 5 in the placebo (18% vs 19%) |
| Bias in measurement of the outcome | LOW | “double-blind” |
| Bias in selection of the reported result | LOW | Results for the outcomes and scales prespecified in the trial registry were available |

Protocol available: <https://clinicaltrials.gov/ct2/show/NCT02385799>

| **Reddihough 2019 (both published data and data retrieved for authors)**  *(Mouti 2014*(71) *– published protocol)* | | |
| --- | --- | --- |
| Methods | Parallel trial (Australia, multi-center) | |
| Participants | 146 children randomised, 71 to placebo and 75 to treatment group (21 discontinued from placebo and 31 from intervention group)  15% F  mean age 11.2 ± 2.9, range 7.5 to 18  Diagnosis: DSM-V criteria for Autism Spectrum Disorder (44.5% Autism, 14.4% Asperger Sdr., 41.1% ASD)  30.1% Intellectual Disability  Required threshold for symptoms: total score at the CYBOCS-PDD of 6 or greater | |
| Intervention | Fluoxetine 16 weeks  starting 4mg/day if <40 kg; 8mg/day if ≥40 kg up to a maximum of 20 mg/d (participants <40 kg) or 30 mg/d (participants ≥40 kg)  Placebo indistinguishable from the active treatment | |
| Outcomes  (**in bold outcomes extracted**)  (*in cursive additional data retrieved from authors*) | **Children’s Yale-Brown Obsessive Compulsive scale – Modified for Pervasive Developmental Disorders - CY-BOCS-PDD**  **Repetitive Behaviour Scale – RBS-R (total score)**  **Spence Children’s Anxiety Scale (total score)**  **Aberrant Behavior Checklist-Community version – ABC-CV (5 subscales)**  **Clinical Global Impression scale- Global Improvement - CGI-I (continuous)**  *Clinical Global Impression scale- Global Improvement - CGI-I (dichotomus)*  Disruptiveness Assessment (created from the authors)  **Adverse events** | |
| Notes |  | |
| ***Risk of bias*** | | |
| **Bias** | **Authors’ judgement** | **Support for judgement** |
| Bias arising from randomization process | LOW | The allocation sequence was a computer-generated randomization schedule created by an independent statistician, using block randomization and stratification. The allocation was concealed (allocation sequence known only by the local pharmacist and indistinguishable medications). However, there are some imbalances at baseline on some relevant behavioural measures, likely to have occurred by chance. |
| Bias due to deviations from intended interventions | LOW | Participants, their families, clinicians, and the research team assessing outcomes remained blinded to the randomization schedule throughout the study.  An appropriate analysis to estimate the effect of assignment to intervention (Modified-ITT (complete case)) was used. |
| Bias due to missing outcome data | HIGH RISK | Very high differential dropout rate (41% in the fluoxetine group vs 29% in the placebo group). For the primary outcome, 25% of participant did not provide data and there is some suggestion that data were not missing completely at random. Secondary analysis (adjusting for baseline imbalances) and multiple imputation analysis lower the difference obtained at the primary analysis below the MID cut-off. |
| Bias in measurement of the outcome | LOW | The method of measuring the outcomes appears appropriate and comparable in the two groups, outcome assessors were blinded to intervention status. |
| Bias in selection of the reported result | LOW | Trial analyzed in accordance with a prespecified plan available both in the trial registry and a published paper. |

Protocol available: <https://www.anzctr.org.au/Trial/Registration/TrialReview.aspx?id=82728>

| **Sugie 2005 (published data only)**  *(Fukuda 2001(52) companion paper - Japanese - published data only)* | | |
| --- | --- | --- |
| Methods | Cross-over (Japan - single center) | |
| Participants | N = 19, 18 completed (1 lost to follow-up); children only  15 boys, 4 girls (21%F)  Mean age 5.4 y, range 3 to 8  Diagnosis: DSM-IV Autism  Threshold for symptoms not mentioned  % of intellectual disability not reported | |
| Intervention | Treatment: Fluvoxamine 12 weeks + 2 weeks tapering down, 2 weeks washout - crossover  Dose: 1 mg/kg/day (for 2 weeks), 2 mg/kg/day (for 3 weeks), 3 mg/kg/day (for 6 weeks), 1.5 mg/kg/day (for 2 weeks) | |
| Outcomes | Behavioural Assessment Scale – BAS (created by the authors)  Childhood Autism Rating Scale - CARS  Clinical Global Impression Scale – CGI  Blood 5-HT level  Adverse events | |
| Notes | Fukuda 2001: effectiveness is not reported by treatment group vs placebo  Sugie 2005: haematological and molecular genetic analysis, effectiveness reported only for genetic subgroup | |
| ***Risk of bias*** | | |
| **Bias** | **Authors’ judgement** | **Support for judgement** |
| Bias arising from randomization process | LOW | Randomly allocated according to a computer-generated list |
| Bias due to deviations from intended interventions | LOW | "Double blind" and carryover effect likely disappeared (2 weeks of washout, fluvoxamine half-life ranges from 19 to 22 hours) |
| Bias due to missing outcome data | LOW | 5.2% missing outcome data: one participant excluded due to non-compliance |
| Bias in measurement of the outcome | LOW | “The prescribing pediatric neurologist, the clinical psychologist who performed the behavioral ratings and […] were unaware of the drug assignment” (double-blind) |
| Bias in selection of the reported result | HIGH | Fukuda 2001: only reported effectiveness for the whole cohort of patients  Sugie 2005: only reported effectiveness for genetic subgroups  Protocol not available |

Protocol not available

| **Greiss Hess 2016 (data retrieved from authors for the subgroup of patients with ASD)** | | |
| --- | --- | --- |
| Methods | Parallel trial (US, Single center) | |
| Participants | 57 children randomised, 30 to placebo and 27 to treatment group (3 discontinued from placebo and 2 from intervention group)  16% F  mean age 3.9 ± 1.1, range 2 to 6  Diagnosis: Fragile-X with or without ASD (DSM-V criteria for of Autism Spectrum Disorder)  (56.1% ASD, 38.6% no ASD, 5.3% missing ASD diagnosis)  % Intellectual Disability not reported  No required threshold for symptoms | |
| Intervention | Liquid sertraline 26 weeks  2.5 mg/day under 4y, 5mg/day 4y and older  Liquid placebo in matching doses | |
| Outcomes  (*in cursive additional data retrieved from authors for the subgroup of patients with ASD*) | *CGI-I (continuous),* *CGI-I (dichotomus)*  Mullen Scales of Early Learning – MSEL  Early Learning Composite – ECL (total and four subscales)  Preschool Language Scales - PLS-5 (Auditory Comprehension, Expressive Communication)  Visual Analog Scale – VAS  Sensory Processing Measure – Preschool - SPM-P  *Adverse Events*  Comprehensive Metabolic Panel and Complete Blood Count | |
| Notes | WE EXTRACTED AND META-ANALYZED DATA ONLY FOR THE SUBGROUP OF PATIENTS WITH DIAGNOSIS OF ASD (~56% of the participants corresponding to 32 patients:15 patients with ASD in the treatment group, 17 patients with ASD in the placebo group) | |
| ***Risk of bias*** | | |
| **Bias** | **Authors’ judgement** | **Support for judgement** |
| Bias arising from randomization process | LOW | “Randomization to sertraline or placebo was carried out  independently by the University of California Davis  Investigational Drug Services.” – no additional details but no apparent baseline imbalances |
| Bias due to deviations from intended interventions | LOW | "Double blind", pre-specified ITT analyses |
| Bias due to missing outcome data | LOW | Missing outcome data for 2 patients in the intervention arm and 3 in the placebo arm.  Considering the subgroup of patients with ASD only one patient in the treatment group early terminated |
| Bias in measurement of the outcome | LOW | “double-blind” |
| Bias in selection of the reported result | LOW | Outcomes and scales prespecified in the trial registry |

Protocol available: https://clinicaltrials.gov/ct2/show/study/NCT01474746

**APPENDIX 4 – LIST OF STUDIES AWAITING CLASSIFICATION**

| **EUCTR2008-003712-36-FR 2008** | |
| --- | --- |
| Methods | RCT |
| Participants | Children with ASD (5-13y) |
| Intervention | Fluoxetine |
| Outcomes of interest | ABC scale  CGI-I  adverse events |
| Notes | Authors attempted to contact investigators |

| **NCT00183339 2005** | |
| --- | --- |
| Methods | RCT |
| Participants | Children with ASD (30-58m) |
| Intervention | fluoxetine |
| Outcomes of interest | Total Score on Caregiver Strain Questionnaire  Aberrant Behavior Checklist Irritability Subscale Score (ABC-I) |
| Notes | Authors attempted to contact investigators |

| **NCT00609531 2008** | |
| --- | --- |
| Methods | RCT |
| Participants | Children and adults with ASD |
| Intervention | citalopram |
| Outcomes of interest | Clinicians Global Improvement Scale  Children’s Yale-Brown Obsessive Compulsive Scale |
| Notes | Authors attempted to contact investigators |
| **NCT00655174 2008** | |
| Methods | RCT |
| Participants | Children with ASD (3-10y) |
| Intervention | Fluvoxamine and sertraline |
| Outcomes of interest | Frequency and severity of autistic behaviours, obsessive symptoms or anxiety |
| Notes | Authors attempted to contact investigators |

| **NCT00787111** | |
| --- | --- |
| Methods | RCT  Open-Label, Follow-On Study of the SOFIA study (Herscu 2019) |
| Participants | Children with ASD (5-18y) |
| Intervention | Fluoxetine |
| Outcomes of interest | CGI  adverse events |
| Notes | Authors attempted to contact investigators |

| **NCT03279471** | |
| --- | --- |
| Methods | RCT  ‘Specifying and Treating Anxiety in Autism Research (STAAR)’ |
| Participants | Children with ASD (8-14y) |
| Intervention | Setraline vs placebo vs CBT/social skills training |
| Outcomes of interest | Pediatric Anxiety Rating Scale (PARS) |
| Notes | Results Submitted to ClinicalTrials.gov on 27.02.2024 – awaiting for quality control review |

| **NCT06081348** | |
| --- | --- |
| Methods | RCT  ‘Sertraline vs. Placebo in the Treatment of Anxiety in Children and AdoLescents With NeurodevelopMental Disorders (CALM)’ |
| Participants | Children with ASD and other neurodevelopmental disorders (8-17y) |
| Intervention | Setraline vs placebo |
| Outcomes of interest | The Screen for Child Anxiety Related Emotional Disorders (SCARED) - parent version  CGI  Adverse events (SMURF)  Pediatric Quality of Life Inventory (PedsQL) |
| Notes | Not yet recruiting |

**APPENDIX 6 - MID ESTIMATES FOR CONTINUOUS MEASURES INCLUDED IN META-ANALYSES OR NARRATIVE SYNTHESES**

| ***Measure*** |  | ***Range of the scale*** | ***10% score change*** |
| --- | --- | --- | --- |
| **Repetitive Behavior Scale-Revised** - RBS-R(72) | Restricted Repetitive Behaviours  43 items (across 6 subscales): behaviors are rated on a 4-point scale (from 0 to 3) | Total range 0 - 129  (lower score better outcomes) | -12.9 points |
| **Preschool Anxiety Scale-Revised** - PAS-R(73) | Anxiety Symptoms  33 items: symptoms are rated from 0 to 4 | 0 – 136  (lower score better outcome) | -13.6 points |
| **Spence Children Anxiety Scale** - SCAS(74) | Anxiety Symptoms  38 items (6 subscales): symptoms are rated from 0 to 3 | 0 - 114  (lower score better outcome) | -11.4 points |
| **Aberrant Behavior Checklist - Community Version - Irritability Subscale** - ABC-CV-I(75) | Irritability and Aggression  15 items: symptoms are rated from 0 to 3 | 0 – 45  (lower score better outcomes) | - 4.5 points |
| **Caregiver Strain Questionnaire** - CSQ(76) | Quality of life (parent reported)  21 items (divided into 3 subscales) - each item is measured using a 5-point scale and a total mean score is calculated | Range 1 to 5  (lower score better outcomes) | -0.5 points (total mean score) |

**APPENDIX 7 - COMPARISON WITH PREVIOUS COCHRANE SYSTEMATIC REVIEW PUBLISHED IN 2013**

|  | **Williams et al. 2013 (27)** | **Current systematic review** |
| --- | --- | --- |
| **Number of studies included** (Pediatric population) | 5 studies:   - **Fenfluramine** (Barthelemy 1989; Leventhal 1993) - **Fluoxetine** (Hollander 2005) - **Fluvoxamine** (Sugie 2005) - **Citalopram** (King 2009) | 7 studies:   - **Fenfluramine** – data not analyzed as it was withdrawn from the market in 1997 - **Fluoxetine** (Hollander 2005, Herscu 2019, Reddihough 2019) - **Fluvoxamine** (Sugie 2005) - **Citalopram** (King 2009) - **Sertraline** (Greiss Hess 2016, Potter 2019) |
| **Total number of participants** **randomized** (Pediatric population) | 240 participants | 631 participants |
| **Outcomes included in meta-analyses** | Including pediatric and adult participants:   - CGI-I | Including only pediatric participants:   - RBS-R - CYBOCS-PDD - ABC-Irritability subscale - CGI-I - Withdrawal due to adverse events - Participants experiencing at least one adverse event |
| **General conclusion** (Pediatric population) | *“There is no evidence of benefit for*  *children, based on one large study of citalopram with low risk*  *of bias and from four smaller studies”* | *“The current body of evidence shows no benefit of the use of SSRIs in*  *children with ASD”* |
