# Supplementary material for: Selective Serotonin Reuptake Inhibitors for Children with Autism Spectrum Disorder: A Systematic Review and Meta-Analysis
Source: Clin Med Insights Pediatr. 2026 Jun 9;20:11795565261442820. doi: 10.1177/11795565261442820 (PMC13254149; doi:10.1177/11795565261442820)
Supplement: sj-docx-2-pdi-10.1177_11795565261442820 – Supplemental material for Selective Serotonin Reuptake Inhibitors for Children with Autism Spectrum Disorder: A Systematic Review and Meta-Analysis [file sj-docx-2-pdi-10.1177_11795565261442820.docx]

| **Section and Topic** | **Item #** | **Checklist item** | **Location where item is reported** |
| --- | --- | --- | --- |
| **TITLE** | | |  |
| Title | 1 | Identify the report as a systematic review. | Selective serotonin reuptake inhibitors for children with Autism Spectrum Disorder: a systematic review and meta-analysis |
| **ABSTRACT** | | |  |
| Abstract | 2 | See the PRISMA 2020 for Abstracts checklist. | Structured abstract (pp. 1–2). The abstract follows PRISMA for Abstracts guidelines, except for funding/conflicts of interest, which are reported in the manuscript body due to the journal’s abstract word count restrictions |
| **INTRODUCTION** | | |  |
| Rationale | 3 | Describe the rationale for the review in the context of existing knowledge. | Background section (pp. 2–6) |
| Objectives | 4 | Provide an explicit statement of the objective(s) or question(s) the review addresses. | Objectives (p. 7) |
| **METHODS** | | |  |
| Eligibility criteria | 5 | Specify the inclusion and exclusion criteria for the review and how studies were grouped for the syntheses. | Methods – Study Selection (p. 9) |
| Information sources | 6 | Specify all databases, registers, websites, organisations, reference lists and other sources searched or consulted to identify studies. Specify the date when each source was last searched or consulted. | Methods – Data Sources and Searches (pp. 8–9) |
| Search strategy | 7 | Present the full search strategies for all databases, registers and websites, including any filters and limits used. | Appendix 1 (full search strategies) |
| Selection process | 8 | Specify the methods used to decide whether a study met the inclusion criteria of the review, including how many reviewers screened each record and each report retrieved, whether they worked independently, and if applicable, details of automation tools used in the process. | Methods – Study Selection (p. 9) |
| Data collection process | 9 | Specify the methods used to collect data from reports, including how many reviewers collected data from each report, whether they worked independently, any processes for obtaining or confirming data from study investigators, and if applicable, details of automation tools used in the process. | Methods – Data Extraction and Quality Assessment (pp. 10–11) |
| Data items | 10a | List and define all outcomes for which data were sought. Specify whether all results that were compatible with each outcome domain in each study were sought (e.g. for all measures, time points, analyses), and if not, the methods used to decide which results to collect. | Methods – Outcomes (pp. 9–10) |
|  | 10b | List and define all other variables for which data were sought (e.g. participant and intervention characteristics, funding sources). Describe any assumptions made about any missing or unclear information. | Methods – Data Extraction (p. 10) |
| Study risk of bias assessment | 11 | Specify the methods used to assess risk of bias in the included studies, including details of the tool(s) used, how many reviewers assessed each study and whether they worked independently, and if applicable, details of automation tools used in the process. | Methods – Data Extraction and Quality Assessment (pp. 10–11); Risk of Bias tool described |
| Effect measures | 12 | Specify for each outcome the effect measure(s) (e.g. risk ratio, mean difference) used in the synthesis or presentation of results. | Methods – Data Synthesis and Analysis (pp. 11–12) |
| Synthesis methods | 13a | Describe the processes used to decide which studies were eligible for each synthesis (e.g. tabulating the study intervention characteristics and comparing against the planned groups for each synthesis (item #5)). | Methods – Synthesis and Analysis (p. 11) |
|  | 13b | Describe any methods required to prepare the data for presentation or synthesis, such as handling of missing summary statistics, or data conversions. | Methods – Data Synthesis and Analysis (pp. 11–12); use of imputation described |
|  | 13c | Describe any methods used to tabulate or visually display results of individual studies and syntheses. | Methods (p. 12); Forest plots & Summary of Findings tables (Figures, Table 1) |
|  | 13d | Describe any methods used to synthesize results and provide a rationale for the choice(s). If meta-analysis was performed, describe the model(s), method(s) to identify the presence and extent of statistical heterogeneity, and software package(s) used. | Methods – Data Synthesis and Analysis (pp. 11–12) |
|  | 13e | Describe any methods used to explore possible causes of heterogeneity among study results (e.g. subgroup analysis, meta-regression). | Methods – Subgroup analysis (p. 12) |
|  | 13f | Describe any sensitivity analyses conducted to assess robustness of the synthesized results. | Methods – Sensitivity analysis (p. 12) |
| Reporting bias assessment | 14 | Describe any methods used to assess risk of bias due to missing results in a synthesis (arising from reporting biases). | Methods – Certainty of the evidence (p. 13); Results – publication bias search mentioned (p. 17) |
| Certainty assessment | 15 | Describe any methods used to assess certainty (or confidence) in the body of evidence for an outcome. | Methods – Certainty of the evidence (pp. 13–14); GRADE tables (Table 1) |
| **RESULTS** | | |  |
| Study selection | 16a | Describe the results of the search and selection process, from the number of records identified in the search to the number of studies included in the review, ideally using a flow diagram. | Results – Study Selection (pp. 14–15); Figure 1 PRISMA flow chart |
|  | 16b | Cite studies that might appear to meet the inclusion criteria, but which were excluded, and explain why they were excluded. | Results – Excluded studies with reasons (p. 15); Appendix 2 |
| Study characteristics | 17 | Cite each included study and present its characteristics. | Results – Characteristics of included studies (p. 16); Appendix 3 |
| Risk of bias in studies | 18 | Present assessments of risk of bias for each included study. | Results – Risk of bias section (pp. 16–18); Figures 2–3; Appendix 3 |
| Results of individual studies | 19 | For all outcomes, present, for each study: (a) summary statistics for each group (where appropriate) and (b) an effect estimate and its precision (e.g. confidence/credible interval), ideally using structured tables or plots. | Results section (pp. 18–25); Forest plots |
| Results of syntheses | 20a | For each synthesis, briefly summarise the characteristics and risk of bias among contributing studies. | Results – beginning of each outcome subsection (pp. 18–25); Table 1 |
|  | 20b | Present results of all statistical syntheses conducted. If meta-analysis was done, present for each the summary estimate and its precision (e.g. confidence/credible interval) and measures of statistical heterogeneity. If comparing groups, describe the direction of the effect. | Results – multiple outcome subsections; Forest plots (pp. 18–25) |
|  | 20c | Present results of all investigations of possible causes of heterogeneity among study results. | Results – limited data prevented subgroup/sensitivity analyses (p. 17) |
|  | 20d | Present results of all sensitivity analyses conducted to assess the robustness of the synthesized results. | Not performed due to insufficient data (p. 17) |
| Reporting biases | 21 | Present assessments of risk of bias due to missing results (arising from reporting biases) for each synthesis assessed. | Results – publication bias search (p. 17) |
| Certainty of evidence | 22 | Present assessments of certainty (or confidence) in the body of evidence for each outcome assessed. | Results – Table 1 (Summary of Findings, GRADE) |
| **DISCUSSION** | | |  |
| Discussion | 23a | Provide a general interpretation of the results in the context of other evidence. | Discussion – Summary of main results (pp. 26–28) |
|  | 23b | Discuss any limitations of the evidence included in the review. | Discussion – Limitations of included evidence (pp. 28–30) |
|  | 23c | Discuss any limitations of the review processes used. | Discussion – methodological considerations (pp. 28–30) |
|  | 23d | Discuss implications of the results for practice, policy, and future research. | Discussion – Implications for practice and future research (pp. 30–33) |
| **OTHER INFORMATION** | | |  |
| Registration and protocol | 24a | Provide registration information for the review, including register name and registration number, or state that the review was not registered. | Abstract and Methods – PROSPERO-CRD42020169836 (p. 1, p. 7) |
|  | 24b | Indicate where the review protocol can be accessed, or state that a protocol was not prepared. | Methods – protocol registered in PROSPERO (p. 7) and publicly available |
|  | 24c | Describe and explain any amendments to information provided at registration or in the protocol. | No amendments were made to the registered protocol |
| Support | 25 | Describe sources of financial or non-financial support for the review, and the role of the funders or sponsors in the review. | Declarations – Funding: none (p. 34) |
| Competing interests | 26 | Declare any competing interests of review authors. | Declarations – Competing interests: none (p. 34) |
| Availability of data, code and other materials | 27 | Report which of the following are publicly available and where they can be found: template data collection forms; data extracted from included studies; data used for all analyses; analytic code; any other materials used in the review. | Declarations – Availability of data and material (p. 34) |

*From:*  Page MJ, McKenzie JE, Bossuyt PM, Boutron I, Hoffmann TC, Mulrow CD, et al. The PRISMA 2020 statement: an updated guideline for reporting systematic reviews. BMJ 2021;372:n71. doi: 10.1136/bmj.n71
